# Suicide in rural Australia: A retrospective study of mental health problems, health-seeking and service utilisation

**DOI:** 10.1371/journal.pone.0245271

**Published:** 2021-07-21

**Authors:** Scott J. Fitzpatrick, Tonelle Handley, Nic Powell, Donna Read, Kerry J. Inder, David Perkins, Bronwyn K. Brew

**Affiliations:** 1 Centre for Rural and Remote Mental Health, University of Newcastle, Orange, Australia; 2 School of Nursing and Midwifery, University of Newcastle, Newcastle, Australia; 3 National Perinatal Epidemiology and Statistics Unit, Centre for Big Data Research in Health and School of Women and Children’s Health, University of New South Wales, Sydney, Australia; University of Toronto, CANADA

## Abstract

**Background:**

Suicide rates are higher in rural Australia than in major cities, although the factors contributing to this are not well understood. This study highlights trends in suicide and examines the prevalence of mental health problems and service utilisation of non-Indigenous Australians by geographic remoteness in rural Australia.

**Methods:**

A retrospective study of National Coronial Information System data of intentional self-harm deaths in rural New South Wales, Queensland, South Australia and Tasmania for 2010–2015 from the National Coronial Information System.

**Results:**

There were 3163 closed cases of intentional self-harm deaths by non-Indigenous Australians for the period 2010–2015. The suicide rate of 12.7 deaths per 100,000 persons was 11% higher than the national Australian rate and increased with remoteness. Among people who died by suicide, up to 56% had a diagnosed mental illness, and a further 24% had undiagnosed symptoms. Reported diagnoses of mental illness decreased with remoteness, as did treatment for mental illness, particularly in men. The most reported diagnoses were mood disorders (70%), psychotic disorders (9%) and anxiety disorders (8%). In the six weeks before suicide, 22% of cases had visited any type of health service at least once, and 6% had visited two or more services. Medication alone accounted for 76% of all cases treated.

**Conclusions:**

Higher suicide rates in rural areas, which increase with remoteness, may be attributable to decreasing diagnosis and treatment of mental disorders, particularly in men. Less availability of mental health specialists coupled with socio-demographic factors within more remote areas may contribute to lower mental health diagnoses and treatment. Despite an emphasis on improving health-seeking and service accessibility in rural Australia, research is needed to determine factors related to the under-utilisation of services and treatment by specific groups vulnerable to death by suicide.

## Introduction

In Australia, suicide rates are higher in rural areas than in major cities, despite similar rates of diagnosed mental disorders [1,2]. Previous studies on rural suicide are limited, focusing on descriptive urban-rural comparisons, small geographical areas, population groups, or a narrow set of predictor variables. A few studies have linked socio-economic inequalities, unemployment, higher alcohol consumption, and limited access to services to elevated suicide risk in rural Australia [3–8]. Geographic clusters of suicide, especially in areas with low access to mental health services and a high degree of socio-economic deprivation, were found to influence metropolitan–regional–remote suicide differentials [5,9,10].

Higher rates of suicide in rural Australia have also been linked with rural traits such as stoicism and low health literacy [11,12]. Such claims mask considerable internal diversity within rural Australia in age, gender and place of residence [13]. Whether higher rates of suicide in these areas derive wholly from specific rural traits such as stoicism or low health literacy, or if rurality merely exacerbates the significant risks associated with suicide such as unemployment, drug and alcohol use, and accessibility of health and social services is unclear [14].

As concern about inequalities in suicide mortality in rural Australia grows, the study of suicide by geographic location is critical to identifying the extent to which the prevalence of suicide is associated with remoteness, thereby enabling better design and targeting of interventions. In this article, we define rural as comprising all areas outside of Australia’s major cities as defined by the Australian Institute of Health and Welfare [2]. In Australia, 29% of the population (around seven million people) live outside of major cities [15]. These Australians often experience poorer health and socio-economic outcomes than people living in major cities [2], and are largely understudied in Australian suicide research. The concept of remoteness is an important aspect of policy development in Australia with service provision influenced by population densities and proximity to urban centres. The Australian Statistical Geography Standard (ASGS) classifies rural areas of Australia into four classes of remoteness based on population sizes and calculated road distances to the nearest service centres [16]. These classes of relative remoteness are:

1. Inner Regional Australia
2. Outer Regional Australia
3. Remote Australia
4. Very Remote Australia

**Fig 1:**
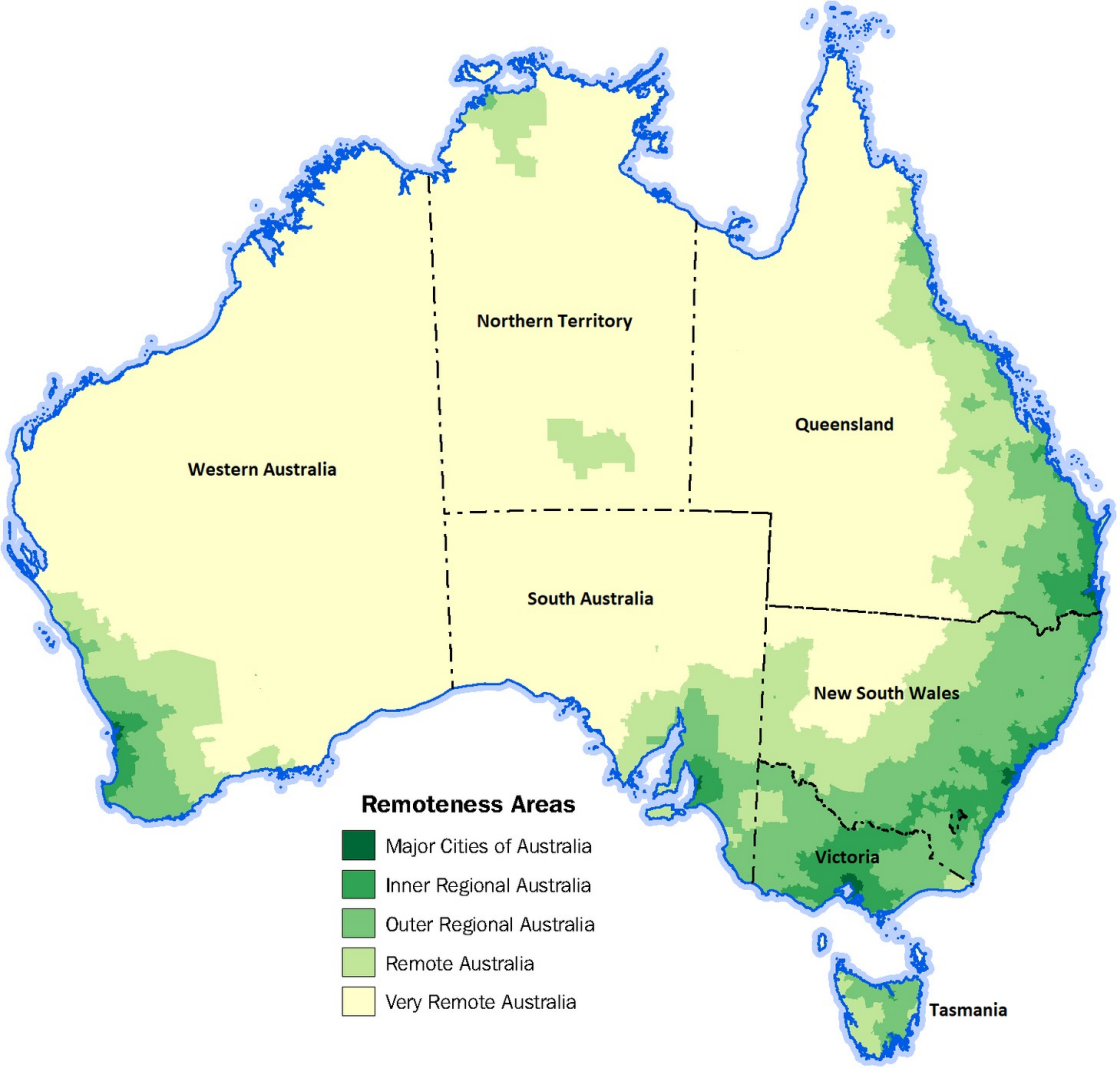
Map of the 2016 remoteness areas for Australia. Based on Australian Bureau of Statistics data [16].

**Fig 1** shows the remoteness structures within Australia. Note that Hobart, the capital city of Tasmania, is classified as inner regional because of the road distance from this city to the closest urban centre located on mainland Australia [17].

## Aims

Using coronial data from four Australian states to obtain a large representative sample of suicide cases from rural Australia, this study aimed to highlight trends, and to examine the prevalence of mental health problems, health-seeking and service utilisation by geographic remoteness for the period 2010–2015.

## Methods

### Ethics statement

The study was approved by the Justice Human Research Ethics Committee (Reference Number CF/16/14124) and The University of Newcastle Human Research Ethics Committee (Protocol Number H-2016-0252). The ethics committees have granted a waiver of consent due to the impracticability of obtaining consent from participants and given sufficient protection of their privacy.

### Sources of data

Data on all closed cases of intentional self-harm deaths by non-Indigenous Australians (hereafter referred to as suicide(s)) in rural New South Wales, Queensland, South Australia and Tasmania for 2010–2015 were extracted from the National Coronial Information System (NCIS). The NCIS is an online repository of Australian and New Zealand coronial data. Coronial data on suicides investigated and identified by Australian and New Zealand coroners in accordance with the International Classification of Diseases, Tenth Revision [18] are transferred to the NCIS for storage and analysis.

NCIS data comprises case records that include demographic information (sex, birth year, postcode of usual residence, marital status, country of birth, indigenous status, period of residency in Australia, employment status and occupation at the time of death) and cause of death details (time, date, location, postcode of incident and death, and mechanism/object/substance causing injury). The NCIS also contains coroner’s findings, autopsy reports, toxicology reports and police reports where available. While all cases have demographic data, not all have additional reports as there are instances where particular procedures are not performed. The availability and quality of reports vary between jurisdictions.

### Data collection

Data were categorised by residential postcode using ASGS Remoteness Area Codes [16]. Data on all closed cases of suicides outside major cities were extracted from the NCIS. Data on Indigenous Australians were not collected due to additional ethical requirements and approval for research involving Indigenous participants that ensures it represents their views and interests.

Toxicology data were obtained from toxicology reports. Alcohol consumption before suicide was assumed if blood alcohol concentrations were ≥ 0.05 g/100mL. This cut-off was selected because it is commonly used in the literature on alcohol use and suicide due to the adverse effects of alcohol on judgement, cognition, mood and behaviour at and above this level [19]. Mental health diagnosis, treatment and service use data were obtained from coroner’s findings, police and autopsy reports. Information in these reports was collected by coroners and police from health records data, and/or evidence provided by treating mental health or medical professionals and next-of-kin. We classified individuals who had mental health symptoms in the absence of formal diagnosis as having ‘undiagnosed symptoms’. SF and DR developed a coding scheme by consensus to systematically capture this information. Regular meetings between SF, DR and NP ensured that coding was consistent across jurisdictions and between coders and that any differences in interpretation or categorisation were resolved collaboratively.

### Statistical analysis

Australian Bureau of Statistics (ABS) 2011 census data were used to determine population numbers by remoteness area for state, age and sex to calculate crude rates of suicide per 100,000 per year where available [20]. The 2011 census data was chosen as it was collected during the study period.

Where general population numbers were not available, proportions per remoteness area category with 95% confidence intervals based on the margin of error were used. The Mantel Haenszel chi-square trend test was used to determine significant linear trends in remoteness [21]. Data management and analysis were performed with IBM SPSS Statistics 25.

### Data completeness and management of missing data

Age, sex, method and socio-economic disadvantage data were available for close to 100% of cases. Marital and employment status data were available for 89.5% of the sample. There were 170 cases (5.4%) with case record data only, and no additional reports (coronial findings, autopsy, toxicology, police reports). The majority of these were in New South Wales (83.5%) (**S1 Table**). Analyses of mental health and health service use data were performed with these 170 cases excluded, leaving 2993 cases for analysis. Two key variables—diagnosis of mental illness and treatment for mental illness—had considerable proportions of missing data (26.2% and 38.0% respectively). To account for this, two sets of analyses were undertaken; a complete case analysis (n = 2993), and a multiple imputation analysis [22]. A fully conditional speculation model was used for the multiple imputation analysis, with ten iterations to replace missing data for each of these variables. Each variable was imputed using age, sex, state, remoteness, marital status and employment status.

## Results

For 2010–2015, there were 3163 closed cases of suicide by non-Indigenous Australians in rural New South Wales, Queensland, South Australia and Tasmania combined. The annual rate for the rural non-Indigenous population in these jurisdictions was 12.7 suicide deaths per 100,000 persons. This was 11.4% higher than the national Australian rate for the same period (11.4 per 100,000 persons) and 15.5% higher than the capital city rate for Sydney, Brisbane and Adelaide combined (11.0 per 100,000 persons). Although the crude number of suicides decreased with remoteness due to smaller populations in remote and very remote areas, suicide rates actually increased by remoteness from 12.5 deaths per 100,000 persons per annum in inner regional areas to 12.7 deaths per 100,000 persons in outer regional areas to 14.0 deaths per 100,000 persons in remote and very remote areas. Rural suicide rates varied by state, from 10.0 deaths per 100,000 persons per annum in New South Wales to 15.5 deaths per 100,000 persons in Queensland. The gradient by remoteness was also more apparent in Queensland (**S2 Table**) The majority of suicides were men (2511 or 79.4%), with an increasing trend by remoteness, from 78.3% of all suicides in inner regional areas to 82.5% of all suicides in remote and very remote areas (p trend <0.05, **Table 2**).

### Demographic characteristics by remoteness

**Fig 2:**
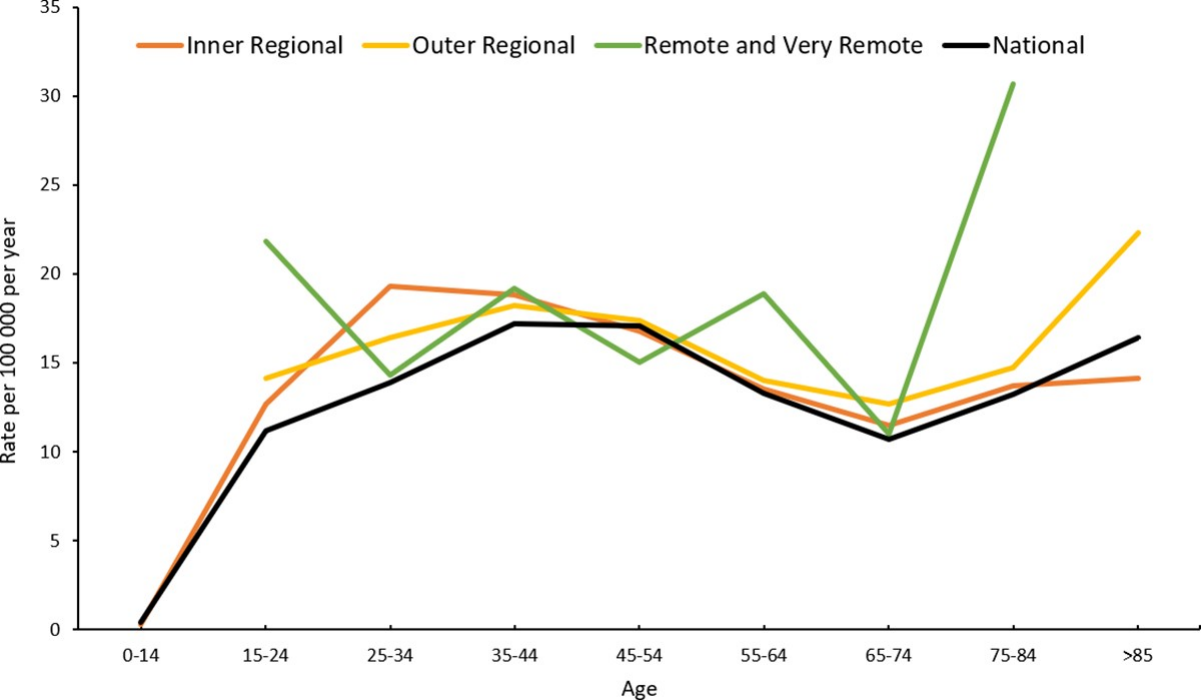
Rural non-Indigenous suicide rates by age and remoteness, 2010–2015, n = 3163.

The demographic characteristics of the sample by remoteness are shown in **Tables 1 and 2**. Different age effects were observed by remoteness, with a higher proportion of suicides in remote and very remote areas occurring in both the younger (15–24 years) and older (75–84 years) ends of the age spectrum (**Table 1, Fig 2**). Concerning marital status, 36.3% of the study population were married or in a cohabiting relationship, 26.8% were never married, and 12.9% were reported as separated. Just over one-third of the study population were employed, one quarter were unemployed, and another quarter were pensioners. Of those employed, 62.1% were tradespersons, technicians, machinery operators or drivers, and labourers; 29.7% were professionals and managers; and 10.0% worked in service industries comprising administration, community and personal services, clerical and retail. Of all suicides, 60.0% were in the lowest two quintiles of Index of Relative Socio-economic Disadvantage (IRSD), while less than 5.0% occurred in the most advantaged quintile. Rates by IRSD were similar across remoteness areas (**Table 2**).

**Table 1:** Rural non-Indigenous suicide cases by age and remoteness, 2010–2015, n = 3163.

	Total		Inner Regional		Outer Regional		Remote and Very Remote		
Age group (years)	n (%)	Rate per 100 000/ year^a^	n (%)	Rate per 100 000/year	n (%)	Rate per 100 000/year^1^	(%)	Rate per 100 000/year^a^	National rate per 100 000/year (Ave 2010–2015)^b^
**0–14**	14 (0.4)	**0.3**	10 (0.5)	**0.3**	<5	**NA**	<5	**NA**	0.4
**15–24**	398 (12.6)	**13.6**	241 (12.2)	**12.7**	130 (12.7)	**14.1**	27 (16.3)	**21.8**	11.2
**25–34**	479 (15.1)	**18.0**	314 (15.9)	**19.3**	143 (14.0)	**16.4**	22 (13.3)	**14.3**	13.9
**35–44**	610 (19.3)	**18.6**	385 (19.5)	**18.8**	194 (19.0)	**18.2**	31 (18.7)	**19.2**	17.2
**45–54**	618 (19.5)	**16.9**	383 (19.4)	**16.8**	208 (20.3)	**17.4**	27 (16.3)	**15.0**	17.1
**55–64**	477 (15.1)	**14.0**	291 (14.7)	**13.5**	156 (15.2)	**14.0**	30 (18.1)	**18.9**	13.3
**65–74**	285 (9.0)	**11.9**	178 (9.0)	**11.5**	96 (9.4)	**12.7**	11 (6.6)	**11.0**	10.7
**75–84**	198 (6.3)	**14.6**	123 (6.2)	**13.7**	60 (5.9)	**14.7**	15 (9.0)	**30.7**	13.2
**>85**	84 (2.7)	**16.6**	49 (2.5)	**14.1**	32 (3.1)	**22.3**	<5	**NA**	16.4
**Total**	3163 (100)	**12.7**	1974 (100)	**12.5**	1023 (100)	**12.7**	166 (100)	**14.0**	11.4

^a^ https://www.abs.gov.au/websitedbs/D3310114.nsf/Home/2016%20search%20by%20geography.

^**b**^ Source: 3303.0- Causes of Death, Australia, 2018, Australian Bureau of Statistics. https://www.abs.gov.au/AUSSTATS/abs@.nsf/DetailsPage/3303.02018?OpenDocument.

**Table 2:** Rural non-Indigenous suicide cases by socio-demographic factors and remoteness, 2010–2015, n = 3163.

	Total (n = 3163)		Inner Regional (n = 1975)		Outer Regional (n = 1023)		Remote and Very Remote (n = 166)		
	n	% (95% CI)	n	% (95% CI)	n	% (95% CI)	n	% (95% CI)	p trend
**Sex**									
Male	2511	79.4 (78.0, 80.8)	1545	78.2 (76.4, 80.0)	829	81.0 (78.6, 83.4)	137	82.5 (76.8, 88.3)	<0.05
Female	652	20.6 (19.2, 22.0)	429	21.7 (19.9, 23.5)	194	19.0 (16.6, 21.4)	29	17.5 (11.7, 23.2)	<0.05
**Marital Status**									
Married/cohabiting	1148	36.3 (34.6, 38.0)	714	36.2 (34.0, 38.3)	367	35.9 (32.9, 38.8)	67	40.4 (32.9, 47.8)	0.58
Never married	847	26.8 (25.2, 28.3)	539	27.3 (25.3, 29.3)	270	26.4 (23.7, 29.1)	38	22.9 (16.5, 29.3)	0.23
Separated	407	12.9 (11.7, 14.0)	259	13.1 (11.6, 14.6)	130	12.7 (10.7, 14.7)	18	10.8 (6.1, 15.6)	0.42
Divorced	272	8.6 (7.6, 9.6)	167	8.5 (7.2, 9.7)	93	9.1 (7.3, 10.9)	12	7.2 (3.3, 11.2)	0.99
Widowed	164	5.2 (4.4, 6.0)	114	5.8 (4.7, 6.8)	46	4.5 (3.2, 5.8)	<5	NA	NA
**Employment Status**									
Employed	1197	37.8 (36.2, 39.5)	759	38.4 (36.3, 40.6)	373	36.5 (33.5, 39.4)	65	39.2 (31.7, 46.6)	0.52
Unemployed	699	22.1 (20.7, 23.5)	419	21.2 (19.4, 23.0)	242	23.7 (21.1, 26.3)	38	22.9 (16.5, 29.3)	0.19
Pensioner	783	24.8 (23.3, 26.3)	503	25.5 (23.5, 27.4)	248	24.2 (21.6, 26.9)	32	19.3 (13.3, 25.3)	0.09
Other^a^	49	1.5 (1.1, 2.0)	37	1.9 (1.3, 2.5)	8	0.8 (0.2, 1.3)	<5	NA	NA
**Index of Relative Socio-Economic Disadvantage**									
Quintile 1	935	29.6 (28.0, 31.2)	584	29.6 (27.6, 31.6)	307	30.0 (27.2, 32.8)	44	26.5 (19.8, 33.2)	0.69
Quintile 2	963	30.4 (28.8, 32.0)	516	26.1 (24.2, 28.1)	395	38.6 (35.6, 41.6)	52	31.3 (24.3, 38.4)	≤0.0001
Quintile 3	658	20.8 (19.4, 22.2)	461	23.3 (21.5, 25.2)	140	13.7 (11.6, 15.8)	57	34.3 (27.1, 41.6)	0.03
Quintile 4	475	15.0 (13.8, 16.3)	310	15.7 (14.1, 17.3)	154	15.1 (12.9, 17.2)	11	6.6 (2.8, 10.4)	≤ 0.01
Quintile 5	127	4.0 (3.3, 4.7)	101	5.1 (4.1, 6.1)	25	2.4 (1.5, 3.4)	<5	NA	NA

^a^ Student/home duties/incarcerated.

### Suicide by method and remoteness

The most common method of suicide was hanging or strangling (49.5%), followed by poisoning (22.4%) and firearm use (12.8%). Firearm use increased with remoteness, from 10.1% in inner regional areas to 24.7% in remote and very remote areas (p trend <0.0001), while poisoning decreased (p trend <0.0001) (**S1 Fig**).

### Suicide and mental health measures by remoteness

**Fig 3:**
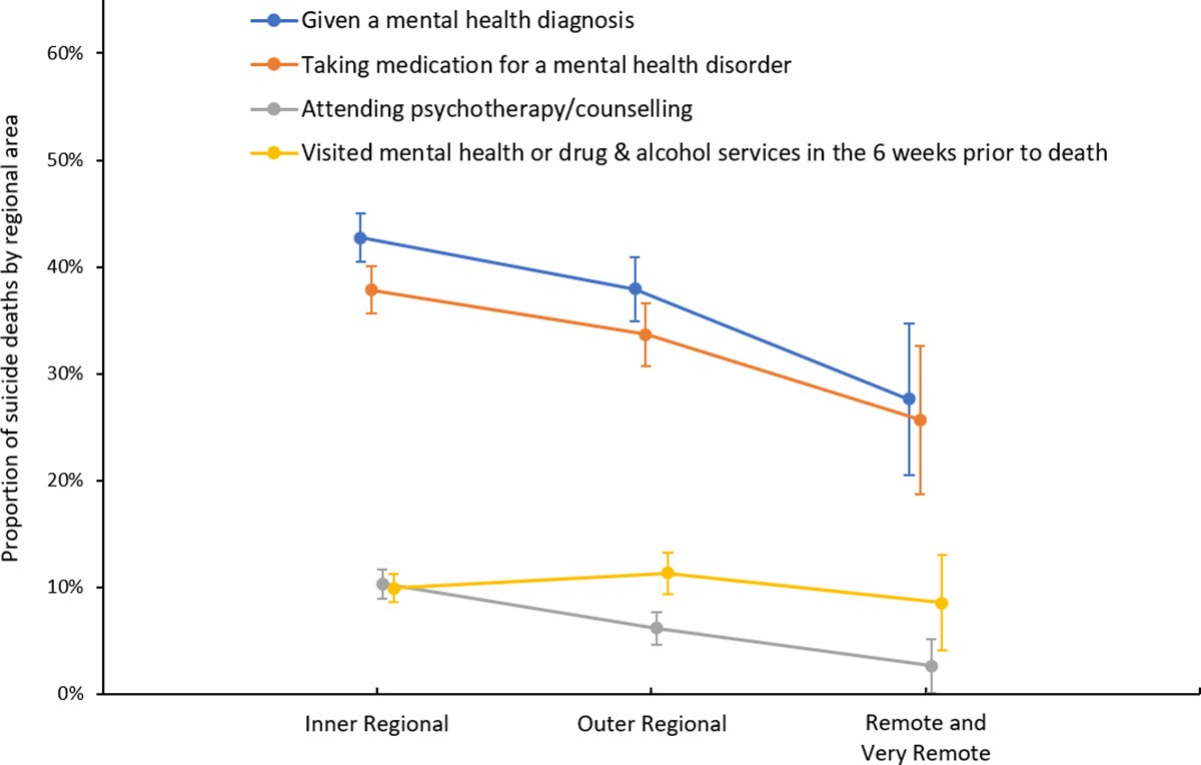
Rural non-Indigenous suicide cases, mental health diagnosis, treatment and health service visits by remoteness, 2010–2015, n = 2993.

In rural areas, 1208 (40.4%) suicide cases had been diagnosed with a mental illness (including substance use disorders), 508 (17.0%) had undiagnosed symptoms of mental illness, 494 (16.5%) had no reported symptoms or mental health problem, and 783 (26.2%) had missing data for mental health (**Table 3**). When missing cases were excluded, this equated to 54.7% of suicides with a diagnosis, 23.0% with undiagnosed symptoms, and 22.4% with no diagnosis. Similarly, when we imputed the missing cases, the multiple imputation analysis indicated 55.7% of suicides had a diagnosis, 23.7% had undiagnosed symptoms, and 20.8% had no recorded mental health issue. Analysis of mental illness by sex (raw data with missing data excluded) found that a higher proportion of females than males had a diagnosed mental illness (64.3% vs. 52.1%, p <0.0001). Analysis by remoteness categories showed a decreasing trend for diagnosis of mental illness across remoteness areas (p trend <0.0001, **Fig 3**). Analysis by sex, however, showed this trend was significant for males (p trend ≤ 0.01) but not females (p trend 0.2).

**Table 3:** Rural non-Indigenous suicide cases and mental health diagnoses by remoteness, 2010–2015, n = 2993.

	Total (n = 2993)	Inner regional n (%) (n = 1852)	Outer regional n (%) (n = 989)	Remote and Very Remote n (%) (n = 152)	p trend
**At least one diagnosis**	1208 (40.4)	791 (42.7)	375 (37.9)	42 (27.6)	<0.0001
**All diagnoses**^**a**^	1483	999	436	48	
**Depression**	918 (61.9)	609 (61.0)	283 (64.9)	26 (54.2)	0.61
**Psychotic disorder**	131 (8.8)	82 (8.2)	39 (8.9)	10 (20.8)	0.05
**Anxiety**	120 (8.1)	86 (8.6)	30 (6.9)	<5	NA
**Bipolar disorder**	118 (8.0)	82 (8.2)	32 (7.3)	<5	NA
**Substance use disorder**	96 (6.5)	76 (7.6)	19 (4.4)	<5	NA
**Personality disorder**	43 (2.9)	29 (2.9)	13 (3.0)	<5	NA
**Trauma-related disorder**	42 (2.8)	29 (2.9)	12 (2.8)	<5	NA
**Other**^**b**^	15 (1.0)	6 (0.6)	8 (1.8)	<5	NA
**Suspected mental health or substance use problem**	508 (17.0)	311 (16.8)	172 (17.4)	25 (16.4)	0.87

^a^ includes cases where more than one diagnosis was given.

^b^ eating disorder/other.

The most common diagnosis was depression, making up 61.9% of all mental illnesses reported, followed by psychotic (8.8%), anxiety (8.1%), bipolar (8.0%) and substance use disorders (6.5%, **Table 3**). Of these cases, almost a fifth (n = 232, 19.2%) had a co-occurring mental illness, with depression and anxiety accounting for 87.1% of all mental health comorbidities. The likelihood of having a substance use disorder decreased with increasing remoteness, while psychotic disorders were increasingly common in remote (RR 2.53, 95% CI: 1.41–4.57) and very remote areas (RR 2.33, 95% CI: 1.24–4.36) compared to inner and outer regional areas respectively. The overall likelihood of receiving a mental health diagnosis decreased with remoteness, although there were no significant trends for depression, anxiety or other psychiatric disorders.

**Fig 4:**
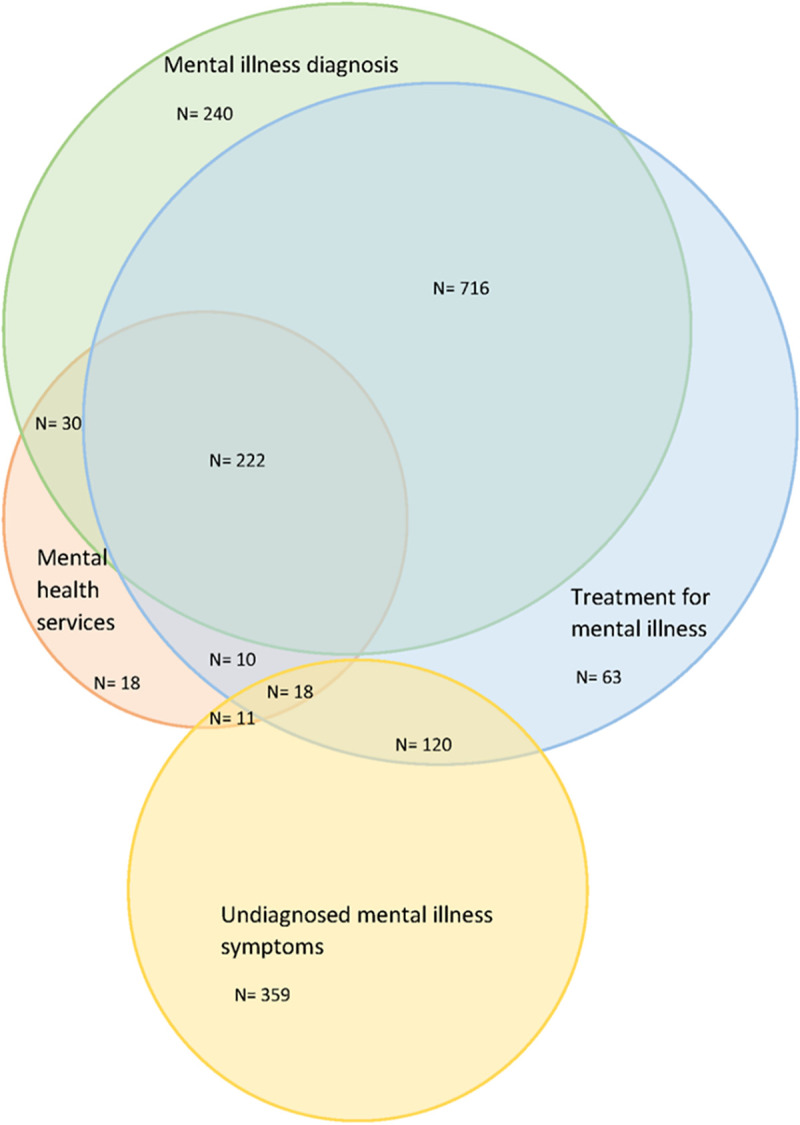
Rural non-Indigenous suicide cases, mental health diagnosis, treatment and mental health service visits in the six weeks before suicide, n = 2993.

In the entire study population, 38.4% (n = 1149) were receiving mental health treatment with medication or psychological therapies. When cases with missing data for mental health treatment were excluded from analysis, this equated to 61.9% of the population. Similarly, the multiple imputation analysis indicated that up to 63.9% of the sample were receiving mental health treatment. Analysis of mental health treatment by sex (raw data with missing data excluded) showed that a higher proportion of females compared with males were receiving treatment (77.0% vs 57.7%, p <0.0001). The majority of those receiving treatment (81.6%) reported a diagnosis of mental illness (**Fig 4**). Similarly, medication and psychological therapies decreased by remoteness with this trend significant for males (p trend ≤ 0.01) but not females (p trend 0.2): medication: from 37.9% in inner regional to 25.7% in remote and very remote areas (p trend ≤ 0.01); psychological therapies: from 10.3% in inner regional to 2.6% in remote and very remote areas (p trend <0.0001) (**Fig 3**). Medication alone was the most common treatment modality reported, accounting for 76.0% of all cases treated. In remote and very remote areas medication alone was reported much more frequently (90.0% of all treated) than in outer (80.3% of all treated) or inner regional areas (73.0% of all treated).

In the six weeks before suicide 22.3% (n = 667) of people had visited a health service at least once, and 6.3% (n = 188) had seen two or more different services (**Table 4**). A further 18.0% had visited a health service in the weeks and months before suicide, but no dates were recorded; hence it is possible that the rates of service attendance in the six weeks before suicide were under-reported. Of all health service visits, 37.5% were to a primary care provider, 37.0% were to a mental health or drug and alcohol service (including community mental health, inpatient mental health, psychiatrist, psychologist), 17.2% visited the emergency department of a hospital, and 8.3% saw other health services (including tertiary and specialist non-mental health, allied health). Proportionately, the number of services visited before suicide was consistent across remoteness areas, except for emergency department visits which declined by remoteness areas. The trend for this association could not be tested, however, because the number of people in remote and very remote areas who visited an emergency department in the six weeks before suicide was less than five. **Fig 4** shows a Venn diagram of the crossover between those in the study population with mental illness diagnoses, those where mental health treatment was reported, those with health-seeking behaviours in the six weeks before suicide, and those with undiagnosed mental health symptoms. This diagram highlights that 19.9% (n = 240) of those with a diagnosed mental illness were not receiving any mental health treatment, nor had they sought specific mental health services (including drug and alcohol services) in the six weeks before suicide. When primary and emergency care data were included in the analysis, the proportion of diagnosed cases not receiving mental health treatment nor seeking health services was 17.5%. For those cases with undiagnosed symptoms, the proportions that were untreated and not seeking health services were even higher: 70.7% (n = 359) not seeking specific mental health services (**Fig 4**) and 65.4% not seeking any health services at all.

**Table 4:** Health service use in the last six weeks before suicide by remoteness, 2010–2015, n = 2933.

Service type	Visits to a health care service in the six weeks before suicide			
	TOTALS n = 2993 n(%)	Inner Regional n = 1852 n(%)	Outer Regional n = 989 n(%)	Remote & Very Remote n = 152 n(%)
**At least one visit to a health service**	667 (22.3)	399 (21.5)	236 (23.9)	32 (21.1)
**At least two visits to a health service**	188 (6.3)	112 (6.0)	63 (6.4)	13 (8.6)
**Total visits (including multiple visits by any one person)**	835	507	287	41
**Mental health or drug & alcohol services (community mental health, inpatient mental health, psychiatrist/psychologist)**	309 (37.0)	184 (36.3)	112 (39.0)	13 (31.7)
**Primary care**	313 (37.5)	187 (36.9)	108 (37.6)	18 (43.9)
**Emergency Department**	144 (17.2)	97 (19.1)	44 (15.3)	<5
**Other (Tertiary Specialist (non-mental health); Allied Health)**	69 (8.3)	39 (7.7)	23 (8.0)	7 (17.0)

### Suicide and toxicology

Toxicology reports showed that 44.5% (n = 1331) of suicide cases had at least one substance (alcohol, illicit drugs or opioids) recorded in their blood at the time of death (**S3 Table**). Blood alcohol readings were present in just over a quarter of suicide cases (25.8%), followed by blood readings for opioids (illicit or prescription) in 13.0%, cannabis in 11.9%, and methylamphetamine in 5.1%. There were no significant increases in evidence of drug use by remoteness except for illicit and prescription opioids which decreased with remoteness from 14.3% in inner regional to 5.3% in remote and very remote (p trend ≤ 0.01, **S3 Table**).

## Discussion

This study examined socio-demographic, mental health and health service use data on all closed cases of suicide by non-Indigenous Australians in rural areas from four Australian states over a five year period. The findings demonstrated that the suicide rate for the rural non-Indigenous population of 12.7 suicide deaths per 100,000 persons was 11.4% higher than the national Australian rate for the same period, consistent with ABS data [23–25]. The suicide rate increased by remoteness, particularly in males.

Remote and very remote areas had peaks in the age distribution of suicide in young people 15–24 years (21.8 per 100,000) and older people 75–84 years (30.7 per 100,000). These findings stand in stark contrast to national suicide rates of persons aged 15–24 and 75–84 years for the same period (**Table 1**). Higher suicide rates among young and older people in rural Australia imply two distinct population groups with likely different contributing factors, necessitating further investigation and targeted suicide prevention strategies.

Findings showed that one-quarter of those who died were unemployed. This was four times higher than the national unemployment rate in 2011 of 5.6% [20]. Unemployment, coupled with decreasing employment opportunities in remote and very remote areas for those with relatively lower skills [26], are likely to increase feelings of distress and despair among specific age-cohorts compared to major cities where there are more possibilities of finding work.

Results from this analysis did not show a difference in suicide trajectories across remoteness areas by disadvantage. This suggests that disadvantage is a significant risk factor for rural suicide irrespective of remoteness [5,27]. However, given the over-representation of disadvantage in smaller towns and geographically isolated locations [28], strategies targeting socially disadvantaged groups, particularly those who are unemployed, may be more beneficial in remote and very remote areas.

Diagnosed mental illnesses were apparent in 40% of those who died by suicide, with an additional 17% displaying symptoms without a formal diagnosis. However, when missing data were first excluded and then imputed, data suggest that the actual rates may be as high as 56% with a formal diagnosis and 24% with undiagnosed symptoms. The high proportion of diagnosed mood disorders is consistent with previous research [29]. Formal diagnoses decreased across remoteness areas, while the presence of symptoms without a diagnosis was consistent. Higher diagnosis of psychotic disorders in remote and very remote areas is unlikely to be related to population prevalence of these disorders as other evidence indicates psychotic disorders are more highly represented in urban environments [30,31]. Instead, it may be explained, in part, by the acute and persistent nature of these disorders and issues relating to the available treatment, care and social supports in remote and very remote areas which sees this population experience higher suicide mortality than in inner and outer regional areas [32,33]. Low levels of substance use disorders in remote and very remote areas are likely to be related to a shortage of drug and alcohol services and specialists, and general practitioners may have less familiarity with problems of addiction than specialist drug and alcohol professionals [34,35].

A proportion of people with diagnosed mental illness or undiagnosed symptoms were not receiving treatment, nor had they sought any health care services in the previous six weeks (17.5% and 65.4% respectively). This represents a potentially vulnerable group whose mental health concerns are not being addressed. Diagnosis and treatment were also higher among females than males. This is consistent with previous research which has shown that women are more likely to have been diagnosed with, and prescribed medication for, mood disorders than men [36]. This is often attributed to men’s reticence to seek help and communicate symptoms [37]. This trend was particularly noticeable in remote and very remote areas where men were less likely to have a diagnosis or to have received treatment than in regional areas. These findings, however, are tempered by the knowledge that 82.5% of those with a diagnosis were either receiving treatment or had sought health care in the six weeks before suicide.

These results point to several concerns. First, they draw attention to the difficulty of identifying high-risk patients and predicting suicidal behaviour in rural areas [38,39]. Second, they suggest that the focus on health-seeking and service accessibility in rural Australia requires rethinking; claims in this regard need to be evaluated in the context of current models of treatment and care that rely heavily on the use of psychiatric medication. Given the established associations between suicide and alcohol use [40], relationship conflict [41], job loss and financial difficulties [42], rural areas may benefit from a generalist model to address the broad range of factors and circumstances that produce considerable depression, anxiety and distress [33,43]. General Practitioners are often required to provide care outside of their clinical speciality. In addition to educational programs, organisational and system-level changes are needed to ensure the quality of care provided to those experiencing mental health concerns is consonant with their needs [44]. Alternative understandings of suicidal distress, coupled with stigma or previous negative experiences of treatment and care, may also reasonably explain the reluctance of those with undiagnosed symptoms to seek help or comply with treatment [45,46].

Treatment for mental illness decreased with remoteness, and there was an increasing trend to rely on medication in remote and very remote areas and less use of psychological therapies. However, there was no decreasing gradient of health service visits in the six weeks before suicide for mental health services (10% of all cases across remoteness) or all health services combined (22.3% of all cases across remoteness). This is broadly consistent with a recent systematic review which found contact rates with mental health services in the month before suicide to be in the range of 7%-32% and primary health care 17%-73% [47]. While unemployment and rurality have been associated with higher levels of pharmacological treatments in comparison to psychological therapies [48], it is less clear why treatment rates decreased by remoteness despite similar rates of service use in this study. However, it may be an indication of lower mental health diagnoses as found in this study, which may be due to the lower availability of mental health specialists in remote and very remote areas. In Australia, psychological services are mostly provided in the private sector and are not viable in remote and very remote areas. Alternatively, socio-demographic, illness or health system characteristics within remote and very remote areas may result in lower treatment adherence [49]. Given the high rates of pharmacological treatments, this may include factors such as privacy, cost, medication information and support, perceptions of illness, or dissatisfaction with treatment choice [50].

Almost half of the cases had a positive toxicology report, and the majority of these were for alcohol. Study findings on the prevalence of alcohol consumption before suicide are consistent with a recent national study [19]. These challenge assumptions about a strong drinking culture in rural Australia. The association between acute alcohol use and impulsive suicide [19], however, does raise additional concerns about the opportunity to intervene, and the importance of selective and indicated suicide prevention strategies that target alcohol or other drug use.

### Strengths and limitations

Suicide in rural Australia is a relatively rare event in an area sparsely populated. The collection of data from four states over multiple years provided opportunities to explore variations in suicide across remoteness areas and over time. This study comprises a unique and comprehensive dataset of suicide in rural Australia. Coding of coronial findings, autopsy and police reports allowed for the analysis of mental health data not available from case records on which the majority of studies analysing coronial data are based. Such information contributes to the identification of factors associated with suicide risk and informs the design and targeting of interventions for rural communities.

This study has some limitations that must be acknowledged. Basic socio-demographic data were available for all cases, however missing or low-quality reports in some jurisdictions meant that 5% or more of data related to mental health, toxicology, and health service visits were not available. In categories where reports were missing (e.g. health service access, mental health diagnosis and treatment) there may be some risk of bias in the results and subsequent analysis. However, multiple imputations of mental illness diagnosis and treatment variables found that rates were similar to those excluding missing data, increasing confidence in the results. We also acknowledge that our results do not fully represent suicide in rural Australia, and that further research is needed to address risk factors specific to Indigenous Australians¸ especially in relation to their historical, cultural and socio-political contexts.

Sample jurisdictions and timeframe for the study were chosen due to resource constraints in securing coronial data from other jurisdictions in the NCIS, and based on consideration of the relatively low numbers of suicide in remote and very remote Australia. As such, the scope of the project did not include data from Western Australia, Victoria or the Northern Territory. While there is no reason to think these states would not follow similar trends, the higher Indigenous populations in Western Australia and the Northern Territory may change some findings. Given that a high proportion of remote communities are Indigenous, together with the high suicide rates among Indigenous Australians [51], a study focusing on Indigenous patterns of suicide by remoteness is highly warranted as a next step in studying the role of remoteness in suicide.

## Conclusion

This study confirms that suicide rates are higher for non-Indigenous Australians in rural than metropolitan areas in Australia, and that suicide rates increase with remoteness. Factors influencing suicide such as socio-economic disadvantage and toxicology were similar by geographic region. However, other elements showed gradients across remoteness such as diagnosis and treatment of mental disorders. The data suggest relatively high rates of mental health problems, service access and treatment. Management of these problems primarily utilized medication despite the association between suicide, unemployment and social disadvantage indicating important implications for service design and policy. The focus on health-seeking and service accessibility in rural areas, while important, may mask the ineffectiveness of current interventions and the reasons for the under-utilisation of services and treatment by specific individuals or groups. Research is needed to determine factors related to the under-utilisation of services and treatment by specific groups vulnerable to death by suicide in rural Australia.

## Supporting information

S1 Fig Rural non-Indigenous suicide cases by method and remoteness, 2010–2015, n = 3163. (TIF)

S1 Table Proportion of rural non-Indigenous suicide cases in National Coronial Information System data from 2010–2015 by state, remoteness and completeness of data, n = 3163. (DOCX)

S2 Table Rural non-Indigenous suicide cases by state, crude numbers and rates /100 000 persons, 2010–2015, n = 3163. (DOCX)

S3 Table Rural non-Indigenous suicide cases and toxicology at the time of death, 2010–2015, n = 2993. (DOCX)
