# Supplementary figures and images for: Suicide in rural Australia: A retrospective study of mental health problems, health-seeking and service utilisation
Source: PLoS One. 2021 Jul 21;16(7):e0245271. doi: 10.1371/journal.pone.0245271 (PMC8294514; doi:10.1371/journal.pone.0245271)

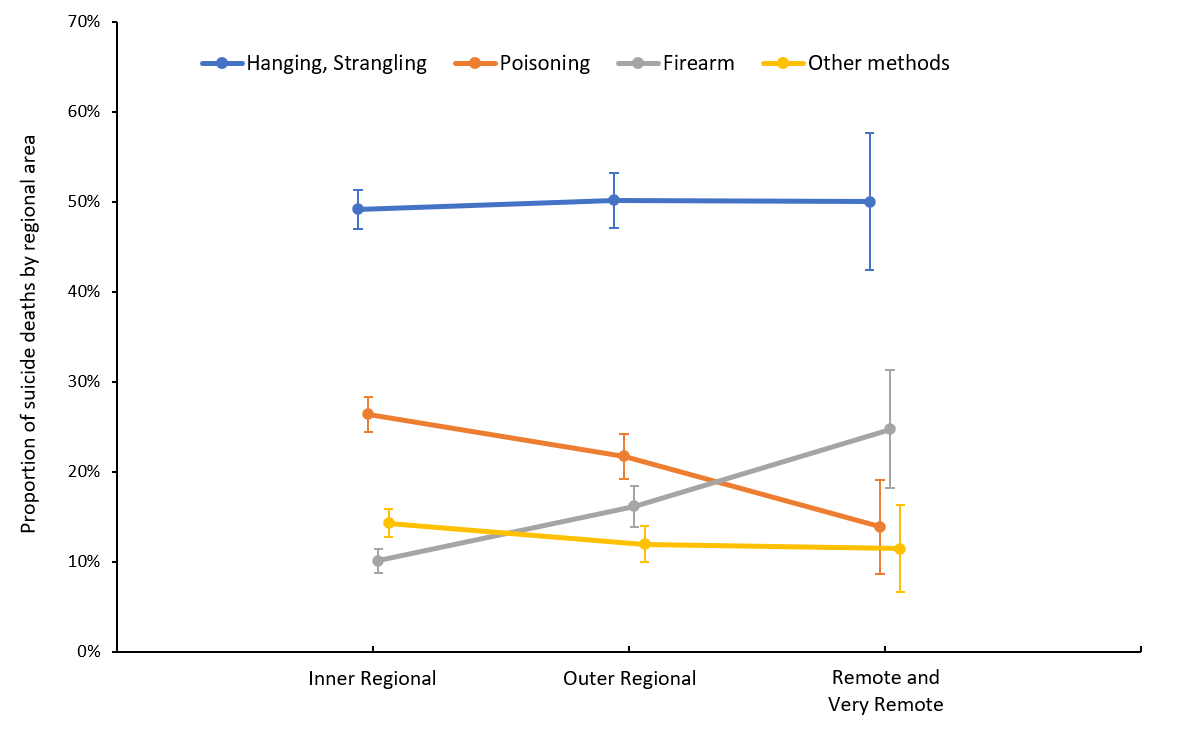

Supplement: S1 Fig — (TIF) [file pone.0245271.s001.tif]
