# Supplementary material for: Suicide in rural Australia: A retrospective study of mental health problems, health-seeking and service utilisation
Source: PLoS One. 2021 Jul 21;16(7):e0245271. doi: 10.1371/journal.pone.0245271 (PMC8294514; doi:10.1371/journal.pone.0245271)
Supplement: S1 Table — (DOCX) [file pone.0245271.s002.docx]

**S1 Table.**

| **Category** | **All suicide cases**  **(n= 3163)**  **n (%)** | **Suicide cases with at least one other report (n= 2993)**  **n (%)** | **Suicide cases with case record data only**  **(n= 170)**  **n (%)** |
| --- | --- | --- | --- |
| New South Wales | 1037 (32.8) | 895 (29.9) | 142 (83.5) |
| Queensland | 1438 (45.5) | 1427 (47.7) | 11 (6.5) |
| South Australia | 310 (9.8) | 293 (9.8) | 17 (10) |
| Tasmania | 378 (12.0) | 378 (12.6) | 0 |
| Inner Regional | 1974 (62.4) | 1852 (61.9) | 122 (71.8) |
| Outer Regional | 1023 (32.3) | 989 (33.0) | 34 (20.0) |
| Remote | 117 (3.7) | 107 (3.6) | 10 (5.9) |
| Very Remote | 49 (1.5) | 45 (1.5) | 4 (2.4) |
