# Supplementary material for: Suicide in rural Australia: A retrospective study of mental health problems, health-seeking and service utilisation
Source: PLoS One. 2021 Jul 21;16(7):e0245271. doi: 10.1371/journal.pone.0245271 (PMC8294514; doi:10.1371/journal.pone.0245271)
Supplement: S2 Table — (DOCX) [file pone.0245271.s003.docx]

**S2 Table.**

|  | **All rural areas**  **(n= 3163)** | | | **Inner Regional**  **(n= 1974)** | | **Outer Regional**  **(n= 1023)** | | **Remote and Very Remote**  **(n= 166)** | | **State average 2010-2015^a^** |
| --- | --- | --- | --- | --- | --- | --- | --- | --- | --- | --- |
|  | Rural population  2011^b^ | n suicides | Rate per 100 000 population per year | n suicides (% of state total) | Rate per 100 000 population per year | n suicides (% of state total) | Rate per 100 000 population per year | n suicides (% of state total) | Rate per 100 000 population per year | **ASR^c^ (95% CI)** |
| **NSW** | 1 725 202 | 1037 | **10.0** | 806 (77.7) | **10.4** | 215 (20.7) | **9.0** | 16 (1.5) | **8.9** | 9.7 |
| **QLD** | 1 539 337 | 1438 | **15.5** | 760 (52.9) | **14.8** | 562 (39.1) | **15.8** | 116 (8.1) | **19.1** | 14.1 |
| **SA** | 415 372 | 310 | **12.4** | 174 (56.1) | **16.9** | 106 (34.2) | **9.3** | 30 (9.7) | **9.4** | 12.8 |
| **TAS** | 474 628 | 378 | **13.3** | 234 (61.9) | **12.4** | 140 (37.0) | **15.5** | <5 | **NA** | 14.2 |

**^a^** Source: 3303.0- Causes of Death, Australia, 2018, Australian Bureau of Statistics. <https://www.abs.gov.au/AUSSTATS/abs@.nsf/DetailsPage/3303.02018?OpenDocument>

^b^ Non-indigenous and not stated population combined <https://www.abs.gov.au/websitedbs/d3310114.nsf/home/about+tablebuilder>
