# Supplementary material for: Suicide in rural Australia: A retrospective study of mental health problems, health-seeking and service utilisation
Source: PLoS One. 2021 Jul 21;16(7):e0245271. doi: 10.1371/journal.pone.0245271 (PMC8294514; doi:10.1371/journal.pone.0245271)
Supplement: S3 Table — (DOCX) [file pone.0245271.s004.docx]

**S3 Table.**

|  | **Total suicides**  **n(%)**  **(n= 2993)** | **Inner Regional**  **n (%)**  **(n= 1852)** | **Outer Regional**  **n (%)**  **(n= 989)** | **Remote and Very Remote**  **n (%)**  **(n= 152)** | **p value** |
| --- | --- | --- | --- | --- | --- |
| At least one substance | 1331 (44.5) | 833 (45.0) | 440 (44.5) | 58 (38.2) | 0.22 |
| Alcohol^a^ | 771 (25.8) | 462 (24.9) | 265 (26.8) | 44 (28.9) | 0.16 |
| Methylamphetamine | 153 (5.1) | 103 (5.6) | 44 (4.4) | 6 (3.9) | 0.14 |
| Cannabis | 356 (11.9) | 216 (11.7) | 124 (12.5) | 16 (10.5) | 0.88 |
| Opioid^b^ | 388 (13.0) | 264 (14.3) | 116 (11.7) | 8 (5.3) | ≤ 0.01 |
| No drugs in bloodstream | 1345 (46.4) | 833 (45.0) | 437 (44.2) | 75 (49.3) | 0.73 |
| Missing | 317 (10.6) | 186 (10.0) | 112 (11.3) | 19 (12.5) | 0.21 |

**^a^** ≥ 0.05 g/100mL

^b^ Illicit and prescription opioids
